# Silver carboxylate-TiO_2_/polydimethyl siloxane is a safe and effective antimicrobial with significant wound care potential

**DOI:** 10.1097/OI9.0000000000000299

**Published:** 2024-03-18

**Authors:** Sai Allu, Colin Whitaker, Benjamin Stone, Neel Vishwanath, Drew Clippert, Elia Jouffroy, Valentin Antoci, Christopher Born, Dioscaris R. Garcia

**Affiliations:** a Warren Alpert Medical School of Brown University, Providence, RI;; b The Diane N. Weiss Center for Orthopaedic Research, Rhode Island Hospital, Providence, RI; and; c Department of Orthopaedic Surgery, Warren Alpert Medical School of Brown University, Providence, RI.

**Keywords:** surgical site infections, silver carboxylate, cytotoxicity, antimicrobial resistance

## Abstract

**Introduction:**

With the rise in antibiotic resistance, new methodologies are needed to combat musculoskeletal infections. Silver is an antimicrobial that can be synthesized in different forms, but its pharmacokinetics are difficult to control. This study details the antibacterial efficacy and cellular cytotoxicity of a formulation consisting of silver carboxylate (AgCar) released through a titanium dioxide/polydimethylsiloxane matrix with a predictable release profile on *Pseudomonas aeruginosa*, *Acinetobacterium baumannii*, *and* human-derived primary osteoblasts.

**Methods:**

Through an Institutional Animal Care and Use Committee and IRB-approved protocol, AgCar was applied to live Yucatan porcine skin and histologically analyzed for skin penetration. Graphite Furnace Atomic Absorption Spectroscopy (GFAAS) was used to measure elution of AgCar. Dose–response curves were generated through optical density to assess potency. Finally, 3-(4,5-dimethylthiazol-2-yl)-2,5-diphenyltetrazolium bromide assay was used to quantify the cellular cytotoxicity of the novel formulation. The results were subject to statistical analysis using analysis of variance and post hoc Tukey tests.

**Results:**

The silver carboxylate coating demonstrated deep penetration into the epithelium at the level of the deep pilosebaceous glands in animal models. GFAAS testing demonstrated the extended elution profile of silver carboxylate over 96 hours, while 100% silver with no titanium dioxide-polydimethylsiloxane matrix fully eluted within 48 hours. 10x silver carboxylate demonstrated superior antimicrobial activity to antibiotics and other silver formulations and showed minimal cytotoxicity compared with other silver formulations.

**Discussion/Clinical Relevance:**

Current antimicrobial therapies in wound care and surgical antisepsis, such as chlorhexidine gluconate, have pitfalls including poor skin penetration and short duration of efficacy. The broad antimicrobial activity, extended elution, and deep skin penetration of this AgCar formulation show great promise for surgical site infection and wound care treatment. Novel technology to fight the growing threat of microbial resistance should be at the forefront of orthopaedic surgical site infection prevention and treatment.

## 1. Introduction

Surgical site infections (SSIs) are one of the primary contributors to surgical morbidity and mortality with a substantial financial impact on the health care system.^1^ The pathogens *Pseudomonas aeruginosa* (*P. aeruginosa*) and *Acinetobacterium baumannii* (*A. baumannii*) are significant contributors to SSIs with increasing resistance to antimicrobials.^2^ An important method for preventing SSIs and wound infections is the application of antimicrobial skin preparations to the site on the skin or the wound.^3^ Most clinically available surgical skin preparations are made up of either chlorhexidine gluconate or povidone-iodine. These preparations have been reported to be subject to reduced susceptibility among both *Staphylococcus Sp.* and *Cutibacterium acnes*.^4,5^ Furthermore, Lee et al^6^ reported that 7 out of 10 of their subjects had positive biopsies for *C. acnes* after preparation with ChloraPrep, due to chlorhexidine gluconate's inability to penetrate pilosebaceous pores deep within the dermis where *C. acnes* tend to colonize.

Owing to the decreasing efficacy of current preventative measures for SSIs and wound infections, silver is becoming an attractive antimicrobial due to its multiple mechanisms of action contributing to its antibacterial properties.^7^ These processes either interfere with bacterial replication or inhibit protein and enzyme function.^8^ The Weiss Center for Orthopaedic Trauma Research has previously characterized and validated a patent-protected antimicrobial coating of silver carboxylate (AgCar) released from a hybrid matrix of titanium dioxide (TiO_2_) and polydimethylsiloxane (PDMS).^9^ This matrix is applied as a liquid and, once dry, uses the ratiometric metal oxide hybrid composition to control the release of AgCar particles.^9^

This study details the antibacterial efficacy and cellular tolerance of silver carboxylate on Multidrug-Resistant *P. aeruginosa*, Multidrug-Resistant *Acinetobacterium baumannii*, and human-derived primary osteoblasts, keratinocytes, and skeletal muscle cells within the context of wound care. We hypothesized that the sustained release of a therapeutic concentration of silver carboxylate from the 95% TiO_2_/PDMS matrix would confer a gentler cytotoxicity profile to primary cells compared with cruder forms of silver and other commonly used last-resort antibiotics, including colisitin, daptomycin, and linezolid.^10^

## 2. Methods

Through an Institutional Animal Care and Use Committee and IRB-approved protocol (PI: Christopher Born, PROTOCOL # 1511000173), 95:5 TiO_2_/PDMS eluting 10x silver carboxylate was applied to live 6″ × 6″ skin sections of the front axillary regions of Yucatan porcine skin using a sponge-tipped applicator. The degree of penetration of silver carboxylate was assessed through histological staining using fast-red and fast-green staining. The thickness of the coating on the skin and pores was determined by using ImageJ (NIH). Graphite Furnace Atomic Absorption Spectroscopy (GFAAS) was used to measure the elution of the coating from polyetheretherketone, a commonly used orthopaedic implant material. To assess potency of silver carboxylate, dose–response curves were generated for *P. aeruginosa* (*P. aeruginosa*) and *Acinetobacter baumannii* (*A. baumannii*) at 1 × 10^7^ CFU/mL and were compared with nanosilver and last-resort antibiotic solutions for 24 hours. The results were measured for viability through optical density (OD) at 570 nm.

Primary human-derived osteoblasts, skeletal muscle cells, and keratinocytes were acquired from ATCC (Manassas, VA) and were each cultured at 1 × 10^4^ cells/mL, 1 × 10^6^ cells/mL, and 1 × 10^4^ cells/mL, respectively, on 96-well plates. After 24 hours, cells were exposed to conditions of 1X silver carboxylate, 10X silver carboxylate, vancomycin (5 µg/mL), vancomycin (50 µg/mL), tobramycin (5 µg/mL), tobramycin (50 µg/mL), linezolid (2 µg/mL), linezolid (20 µg/mL), Polymyxin E (2 µg/mL), 10 nM silver nanoparticles, 30 nM silver nanoparticles, 100 nM colloidal silver, and 300 nM colloidal silver. A media blank, a cell blank, and a 95% TiO_2_/PDMS matrix-only condition were used as negative controls, and 100% silver carboxylate and 1% Triton X were used as positive controls. Silver carboxylate conditions, TiO_2_/PDMS only negative control, and 100% silver carboxylate with no matrix positive control were added by dip coating 2-mm glass beads. After 24 hours, cell viability was measured by PR omega Cell Titer 96 NonRadioactive Cell Proliferation Assay protocol (3-(4,5-dimethylthiazol-2-yl)-2,5-diphenyltetrazolium bromide [MTT] assay) and spectrophotometry at a wavelength of 570 nm. OD of each condition was then compared with OD of the cell blank to determine percent cell viability.

## 3. Results

**Figure 1.**
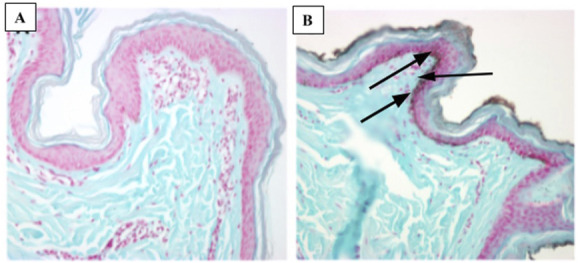
Silver carboxylate penetrates deep into the pilosebaceous glands (Yucatan pig skin penetrance assay) (A) control (B) silver carboxylate.

**Figure 2.**
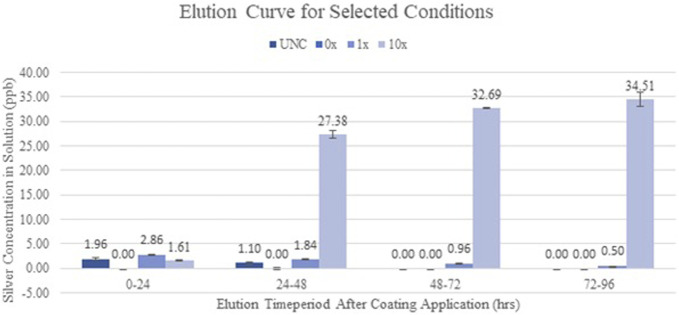
Silver carboxylate elutes curve for UNC, 0x, 1x, and 10x PEEK implants. The 10X AgCar condition maintained a consistent, significantly greater elution through the 96-hour period compared with 1X AgCar condition. The 0X condition eluted undetectable low levels of AgCar. PEEK, polyether ether ketone.

**Figure 3.**
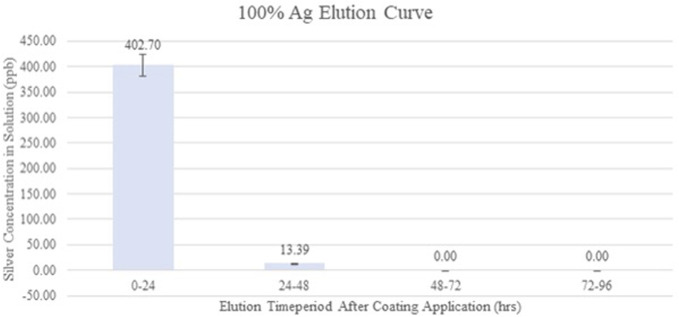
Silver carboxylate elution curve for 100% Ag PEEK implants. The 100% AgCar condition eluted a large amount of AgCar in the first 24-hour period but did not maintain the rate of elution during the rest of the study period. There were undetectable levels of AgCar eluted during the 48–96-hour period. PEEK, polyether ether ketone.

The porcine skin data have demonstrated that silver carboxylate will penetrate deep into the epithelium and is seen at the level of the deep pilosebaceous glands (Fig. 1). The GFAAS data (Figs. 2 and 3) demonstrate the elution profiles of the silver carboxylate coatings at its various concentrations after the coated polyether ether ketone implants were allowed to elute throughout 24-hour periods, for a total of 96 hours. The 0x implants demonstrated undetectably low levels of AgCar for every 24-hour period examined (Fig. 2). By contrast, the 1x implant eluted 2.86 ppb the first day and decreased by 35%–50% for each subsequent day (Fig. 2). The 10x condition eluted 1.61 ppb of AgCar during the first 24 hours. After the first 24 hours, AgCar concentrations rose by 1600% to 27.38 ppb and proceeded to increase slightly through the subsequent days (Fig. 2). On the other hand, the 100% Ag coating demonstrated a high initial elution of 402.70 ppb during the first 24 hours, with undetectable levels of AgCar in the solution after 48 hours (Fig. 3). This suggested that all particles had been fully eluted from the 100% Ag solution in the first 48 hours (Fig. 3).

**Figure 4.**
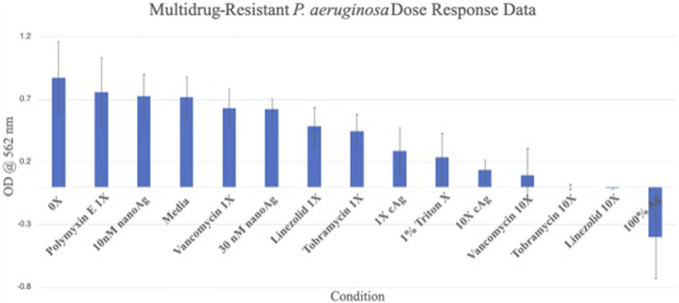
Dose response data for MDR *P. aeruginosa*. Both the 1X and 10X AgCar conditions were more efficacious against MDR *P. aeruginosa* than all 1X antibiotic concentrations but less effective than the 10X antibiotic concentrations.

**Figure 5.**
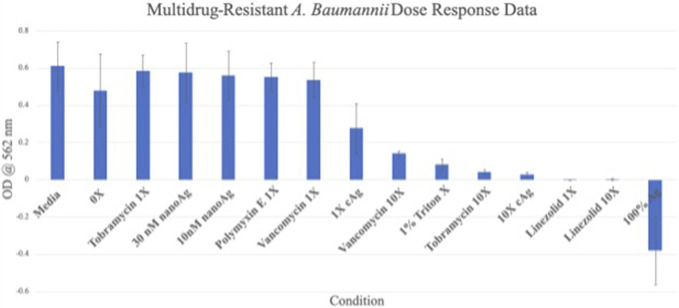
Dose response data for MDR *A. Baumannii*. The 10X AgCar condition is more efficacious against MDR *A. Baumannii* compared with all the last-resort antibiotics and other silver formulations, except for 1X and 10X linezolid concentrations.

The dose response data showed that both the 1X (OD = 0.289 ± 0.181) and 10X (OD = 0.135 ± 0.082) silver carboxylate conditions were more efficacious against multidrug resistant (MDR) *P. aeruginosa* than all 1X antibiotic concentrations but less effective than the 10X antibiotic concentrations (Fig. 4). When tested against MDR *A. baumannii,* the data show similar results. The 1X (OD = 0.276 ± 0.135) and 10X (OD = 0.026 ± 0.014) silver carboxylate conditions were more efficacious against most of the antibiotic concentrations except the 1X and 10X linezolid concentrations (Fig. 5). Both of the silver carboxylate conditions demonstrated a similar antimicrobial profile to the last-resort antibiotics and cruder forms of silver, when tested against MDR *P. aeruginosa* and MDR *A. baumannii*.

**Figure 6.**
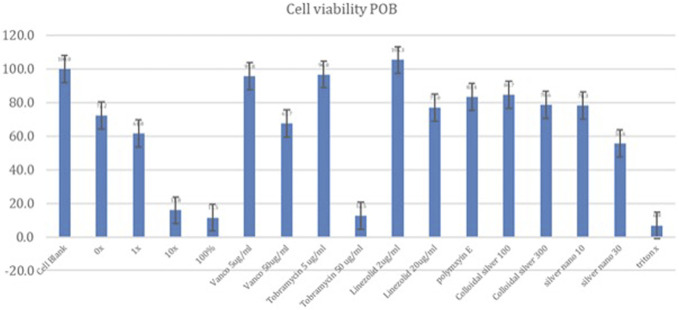
Primary osteoblast (POB) cell viability. The 1X silver carboxylate condition demonstrated cell viability that was comparable with linezolid (20 µg/mL) and greater than tobramycin (50 µg/mL). Osteoblasts were sensitive to the 10X silver carboxylate condition, demonstrating cytotoxicity that was similar to the positive controls (100% silver carboxylate and triton X).

Osteoblasts were sensitive to silver carboxylate yielding the following viabilities relative to the cell blank (Fig. 6): 95% TiO_2_/PDMS matrix-only (72.2%), 1X silver carboxylate (61.8%), 10X silver carboxylate (15.8%), and 100% silver carboxylate (11.5%). Against antibiotics, osteoblasts were less sensitive, showing sensitivity of 95.8% against vancomycin (5 µg/mL), 67.7% against vancomycin (50 µg/mL), 105.3% against linezolid (2 µg/mL), 77.0% against linezolid (20 µg/mL), 96.7% against tobramycin (5 µg/mL), 12.5% against tobramycin (50 µg/mL), and 83.4% against Polymyxin E (2 µg/mL). Against crude formulations, osteoblasts showed the following results: silver nanoparticles (10 nM) (78.3%), silver nanoparticles (30 nM) (55.6%), colloidal silver (100 nM) (84.6%), and colloidal silver (300 nM) (78.6%).

**Figure 7.**
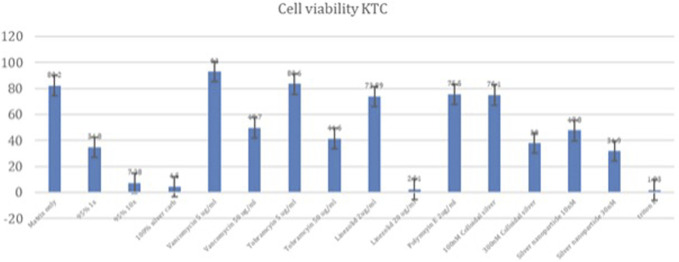
Keratinocyte (KTC) cell viability. The 1X silver carboxylate condition demonstrated cell viability that was comparable with vancomycin (50 µg/mL) and tobramycin (50 µg/mL) and greater than linezolid (20 µg/mL). Keratinocytes were sensitive to the 10X silver carboxylate condition, demonstrating cytotoxicity that was similar to the positive controls (100% silver carboxylate and triton X).

Skeletal muscle cells showed the following cell viabilities relative to the cell blank (Fig. 7): 95% TiO_2_/PDMS matrix only (107%), 1X silver carboxylate (88.6%), 10X silver carboxylate (16.7%), and 100% silver carboxylate (14.8%). When treated with antibiotics, skeletal muscle cells showed overall lower cell viabilities, with a viability of 99.1% when treated with vancomycin (5 µg/mL), 80.4% when treated with vancomycin (50 µg/mL), 22.8% when treated with linezolid (2 µg/mL), 0.77% when treated with linezolid (20 µg/mL), 82.0% when treated with tobramycin (5 µg/mL), 57.1% when treated with tobramycin (50 µg/mL), and 0.70% when treated with Polymyxin E (2 µg/mL). When treated with crude silver formations, skeletal muscle cells showed the following results: silver nanoparticle (10 nM) (97.3%), silver nanoparticle (30 nM) (63.5%), colloidal silver (100 nM) (86.1%), and colloidal silver (300 nM) (89.3%)

**Figure 8.**
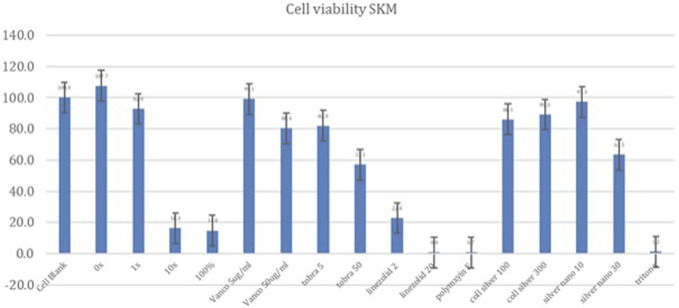
Skeletal muscle (SKM) cell viability. The 1X silver carboxylate formulation resulted in a higher cell viability compared with all the antibiotics tested except for vancomycin (5 µg/mL). Skeletal muscle cells were sensitive to the 10X silver carboxylate condition, demonstrating cytotoxicity that was similar to the positive controls (100% silver carboxylate and triton X).

Keratinocytes showed the following cell viabilities relative to the cell blank (Fig. 8): 95% TiO_2_/PDMS matrix only (82.2%), 1X silver carboxylate (34.8%), 10X silver carboxylate (7.29%), and 100% silver carboxylate (4.50%). When treated with antibiotics, keratinocytes showed a viability of 93.0% when treated with vancomycin (5 µg/mL), 49.7% when treated with vancomycin (50 µg/mL), 73.9% when treated with linezolid (2 µg/mL), 2.50% when treated with linezolid (20 µg/mL), 83.5% when treated with tobramycin (5 µg/mL), 41.5% when treated with tobramycin (50 µg/mL), and 75.5% when treated with Polymyxin E (2 µg/mL). When treated with cruder silver formations, keratinocytes showed the following results: silver nanoparticle (10 nM) (47.8%), silver nanoparticle (30 nM) (31.8%), colloidal silver (100 nM) (75.1%), and colloidal silver (300 nM) (38.0%)

## 4. Discussion/conclusions

Current antimicrobial therapies in wound care and surgical antisepsis, such as chlorhexidine gluconate, have pitfalls including poor skin penetration and reduced efficacy, particularly in the face of antimicrobial resistance.^5^ However, the porcine skin data have demonstrated that silver carboxylate will penetrate deep into the epithelium, particularly in the pilosebaceous pores. Furthermore, the GFAAS data have demonstrated consistent elution from the 1X and 10X silver carboxylate coating through 96 hours. By contrast, the 100% Ag conditions showed a very large, immediate release of AgCar in the first 24 hours with no further release during the following 3 days. This confirms the importance of the TiO_2_-PDMS matrix for the controlled release and consistent efficacy of AgCar. The dose-response curves establish that the antimicrobial efficacy of the 1X and 10X silver conditions resemble that of last-resort antibiotics including vancomycin, linezolid, Polymyxin, and tobramycin. This coating's ability to penetrate into the dermis, maintain a consistent rate of elution, and demonstration of an antimicrobial profile similar to that of last-line antibiotics make it a promising therapeutic consideration. However, the cytotoxicity profile of any antimicrobial treatment must be established before it can be contemplated for treatment in humans in the setting of SSIs and wound care.

The safety profile of silver carboxylate was characterized by performing the MTT cell viability assay for each of the human cell lines. The MTT cell viability assay yielded mixed results. While osteoblasts were sensitive to silver carboxylate, the cytotoxicity profile of the silver carboxylate conditions were less severe than those for many of the commonly used last-resort antibiotics and cruder forms of silver. In osteoblasts, the 1X silver carboxylate showed 61.8% viability while the 100% silver carboxylate (no TiO_2_/PDMS matrix) demonstrated 11.5% viability (Fig. 6). This large difference in viability is observed in each of the cell lines and can be explained by the decrease in the toxicity of silver carboxylate when combined with the titanium dioxide/PDMS matrix, which permits controlled release of silver ions. Furthermore, 1X silver carboxylate showed comparable safety to linezolid (20 µg/mL) and vancomycin (50 µg/mL) and superior safety to tobramycin (50 µg/mL) in osteoblasts. When applied to keratinocytes, silver carboxylate 1X resulted in cell viability that was comparable with the cell viability observed in higher concentrations of antibiotics, specifically vancomycin (50 µg/mL) and tobramycin (50 µg/mL). Superior cell viability was observed compared with linezolid (20 µg/mL) (Fig. 7). Of all 3 cell lines, skeletal muscle cells showed the highest cell viability when treated with silver carboxylate. The 1X silver carboxylate formulation resulted in a higher cell viability compared with all the antibiotics tested except for vancomycin (5 µg/mL) (Fig. 8).

Previous studies have characterized the cytotoxicity of silver carboxylate in fibroblasts and epithelial ovarian cancer cells, showing minimal cytotoxic effects.^11^ Moreover, Tran et al^12^ performed WST-1 viability assays in osteoblasts and demonstrated a concentration sufficient to exhibit bactericidal effects against *Staphylococcus aureus* without significantly reducing osteoblast growth. The MTT data from this study aid in expanding the safety profile of silver carboxylate in additional human cell lines. This safety profile is encouraging for the clinical application of silver carboxylate at the 1X concentration to bony wounds and repairs, where osteoblast and skeletal muscle cell activity predominates.

Silver carboxylate has demonstrated efficacy at the 1X concentration against *P. aeruginosa* and *A. baumannii* (Figs. 4 and 5). This study provided evidence of silver carboxylate's antimicrobial activity against multidrug-resistant pathogens and low toxicity against osteoblasts. These findings in combination with the deep penetration of the novel silver carboxylate-TiO_2_/polydimethyl siloxane coating into pilosebaceous glands make this a very promising chemistry for therapeutic treatment.

## 5. Clinical relevance/future directions

Novel technology to fight the growing threat of microbial resistance should be at the forefront of orthopaedic surgical site infection prevention and treatment. The broad antimicrobial activity, extended elution, and deep skin preparation of silver carboxylate demonstrated by the porcine skin data show great promise for SSI and wound care treatment. Further research needs to be conducted to assess the full antimicrobial profile against additional pathogens that are common etiologic agents for surgical site infections and for wound care. The low cytotoxic profile of 1X silver carboxylate further supports the use of this compound in vivo, particularly in synergy with other antimicrobials, which could extend the length of efficacy of these compounds. To further characterize the safety of this coating, the cytotoxicity profile of silver carboxylate will be established in endothelial cells—a cell line critical to the formation of granulation tissue in wound healing.
